# Virtual Learning in Obstetrics and Gynecology (VLOG): A Four-Day Distance Learning Course for Clerkship-Level Medical Students

**DOI:** 10.7759/cureus.83471

**Published:** 2025-05-04

**Authors:** Rene MacKinnon, Jill Brown, Elizabeth Dunn, Joy Wheat, Katerina Shvartsman

**Affiliations:** 1 Gynecologic Surgery and Obstetrics, Uniformed Services University of the Health Sciences, Bethesda, USA; 2 Gynecologic Surgery and Obstetrics, Naval Medical Center Portsmouth, Portsmouth, USA

**Keywords:** clerkship, curriculum, distance learning, medical student, obstetrics and gynecology

## Abstract

Objective: The COVID-19 pandemic created unique challenges for clinical medical education, as students and educators had to pivot to primarily distance learning. We aimed to develop a novel curriculum to engage students in continued learning and maintain clinical readiness.

Design: To substitute for in-person Obstetrics and Gynecology (OBGYN) clerkship teaching during the pandemic, we created a structured, multi-media virtual course utilizing reading materials, podcasts, and videos for self-paced, student-controlled learning supplemented by virtual group discussions with faculty. Following course completion, students completed surveys to assess their attitudes, preferences, and concerns regarding this virtual curriculum.

Setting: This program was conducted at the Uniformed Services University School of Medicine.

Participants: Clerkship-level medical students at the Uniformed Services University were included in the program.

Results: Ninety-eight percent of the survey respondents reported that the curriculum allowed for individualization of their education; 96% of learners reported that the course improved their fundamental understanding of OBGYN topics; and 88% indicated improved readiness to start or resume their core clerkship after this course.

Conclusion: A self-paced multi-media approach to medical student OBGYN education allowed for individualized learning and was overall well received. Although this program was designed in response to the COVID-19 pandemic, it can be utilized as an introductory module for the OBGYN clerkship or to fill gaps for students who may miss clinical time. This course template can be adapted for other specialties.

## Introduction

In March 2020, the AAMC released a statement advising that medical students be withdrawn from clinical rotations due to the COVID-19 pandemic [1]. While healthcare workers continued to battle their newest and possibly greatest challenge, many medical students were forced to halt their clinical education abruptly. One report found that among 200 medical schools in the United States, three-quarters suspended or canceled their clinical activities for third- and fourth-year medical students [2]. This suspension of clinical teaching was unlike other national disasters, where students have historically been allowed opportunities to participate in healthcare, for example, after hurricanes, tropical storms, and wildfires [3].

Traditionally, medical education consists of two years of didactic teaching followed by two years of face-to-face clinical work. Although this paradigm has evolved over time, with many institutions emphasizing earlier clinical exposure, the overall structure of “lecture years” followed by “clinical rotation years” has been generally maintained. What has dramatically changed is the innovation and incorporation of technology in medical education. Digital platforms to record lectures and allow individualization of learning have become increasingly popular in medical schools over the last several years. However, this individualized, efficient, and flexible strategy has primarily been applied to the early didactic years of the medical school curricula [3]. Until the safety of students was compromised by the COVID-19 pandemic, medical education had never been forced to improvise a clinical curriculum.

Several articles during this educational transition called to action this overdue change in medical education, describing the COVID-19 pandemic as a turning point in changing how we teach learners [2-12]. Examples include allowing students to learn at their own pace through recorded lectures, where students could pause to take notes and emphasize their personalized take-home points [4]. The “flipped classroom” model focuses on the interaction between teachers and learners by having students complete pre-work before class. Compared to traditional didactic learning, this style of education has been shown to promote higher-order thinking and student satisfaction [13,14]. Although this structure is more easily applicable to early medical school “didactic” students, it also applies to how we teach lectures, especially as problem- and case-based teaching remains the cornerstone of interactive learning [3]. Furthermore, implementing technology into medical education encourages students to adapt to new teaching styles and navigate these unchartered waters. In theory, students benefit from technology-driven interactive lessons not only educationally but also personally and professionally by practicing adaptability, teamwork, and improvisation [5].

At the onset of the pandemic, the Obstetrics and Gynecology (OBGYN) clerkship at the Uniformed Services University had nine rotation sites throughout the country, spanning from Hawaii to the East Coast. As our clerkship students were instructed to return to the University, clerkship directors were challenged to quickly fill this academic void. The OBGYN clerkship faculty sought to create a course aimed at developing clinical skills and knowledge through distance learning. We aimed to design an innovative, technology-driven, and interactive curriculum to allow the personalization of lessons while preparing students to return to or start their OBGYN clinical rotations.

## Materials and methods

Clerkship directors from six core subspecialties (surgery, internal medicine, pediatrics, psychiatry, family medicine, and OBGYN) at the Uniformed Services University were tasked with creating a program to engage clerkship students who were pulled from their clinical rotations due to the COVID-19 pandemic. In response to this request, the clerkship directors developed a scaffolding for a four-week course titled the “COVID Clerkship Interim Module” (CCIM). Within this framework, each clerkship director was allotted 3-4 days to design a clinical-based curriculum. 

The OBGYN clerkship was allocated four days to deliver a clinically relevant curriculum focused on fundamental topics. We developed a four-day, self-paced, self-study course supplemented with interactive group discussions that took place on a virtual platform. All 133 students who had not completed their OBGYN rotation were enrolled in this course. Following completion, we evaluated students’ attitudes and perspectives on this improvised distance-learning curriculum. This study was deemed exempt by the Uniformed Services University Institutional Review Board. The learners who completed this four-day interim course eventually completed their five-week in-person clerkship within the academic year.

The curriculum design and course objectives were based on 13 core OBGYN topics derived from the Association of Professors of Gynecology and Obstetrics (APGO) medical student educational objectives, including history and physical exam, cervical cancer screening, preventive care and health maintenance, intrapartum care, intrapartum fetal surveillance, abnormal labor, postpartum hemorrhage (PPH), contraception/family planning, sexually transmitted infections, ectopic pregnancy, normal and abnormal uterine bleeding, uterine leiomyoma, and gynecologic procedures [15]. These topics were considered representative of common patient concerns and provided a solid foundation for clinical problem-solving. 

Once the core topics were identified, the clerkship team researched various educational options, including relevant videos, book chapters, case scenarios, podcast episodes, and procedure descriptions/steps to create a multi-media curriculum that would appeal to various student learning styles. All educational components were open-source online or available to students through our library. The sample resources used are given in Table 1. The resources recommended were vetted by the curriculum development team to ensure reputable content was provided through professional organizations and reliable educational platforms or textbooks. Additionally, we estimated how long each activity would take to complete to ensure students had adequate time to accomplish the course objectives in the period allotted. Furthermore, to help solidify knowledge, each topic was associated with a set of standardized questions from the UWise© question bank (our department subscribes to this question bank through APGO.org). 

**Table 1:** Sample resources

Videos	APGO medical student educational objective videos
Podcast	The OBG Med Student Podcast
Book	Obstetrics and Gynecology by Beckmann and Ling, 9th Edition
Teaching cases	APGO Medical Student Educational Objectives Videos
Procedures	ACOG.org Links: https://stanfordmedicine25.stanford.edu/the25/PelvicExam.html; https://www.merckmanuals.com/professional/pages-with-widgets/procedures-and-exams?mode=list; https://www.innovating-education.org/
Mobile apps	CDC Contraception; WHO Contraception; CDC STI Tx Guide; ASCCP management guidelines for cervical screening
Other	ACOG.org – Practice Bulletins; Procedure Ready Ob/Gyn Podcast; Procedure videos on YouTube

At the end of days 2 and 4, which included a total of six hours of self-paced learning through independent resource options and practice questions, students participated in one-hour, virtual, small-group case discussions facilitated remotely by faculty and resident physicians from around the country. Faculty were recruited via email from across the military health system. Students were grouped by their assigned clerkship site, with approximately 10 students in each group. We attempted to pair students with faculty at their future rotation site to facilitate familiarity with a teacher they may work with in the future. There was a total of 14 groups, and each small group was assigned a unique Google Meet link. Cases were derived from the APGO Medical Student Educational Objectives and included one obstetrical case (PPH) and one gynecological case (gynecologic procedures) [7]. Virtual office hours were offered throughout the course. Students resumed their clinical clerkship schedule approximately one month after completing the distance learning course. 

After completing the distance-learning OBGYN course, students were given two anonymous questionnaires using Likert-scale and free-response options via Google Forms©. The OBGYN clerkship team created the survey questions to help evaluate course effectiveness and gather feedback for future improvement. The first survey, referred to as the “post-course survey,” was distributed immediately after the completion of the course. The second survey was distributed to each student after they completed their in-person OBGYN rotation. This is referred to as the “post-clerkship survey.” The post-course survey asked free-response questions such as “What did you enjoy about this course? What were the strengths of the course?” and “What improvements would you recommend for this course?” The post-clerkship survey included free-response questions such as “What did you find most helpful about the course now that you have completed your clerkship?” and “What did you find the least helpful about the course?”

The survey data and qualitative responses were analyzed using Google Forms©. To evaluate the data from free-response questions, the authors assessed themes from responses, with the goal of highlighting commonalities across learner experiences.

## Results

A total of 133 students completed the four-day virtual course. The post-course and post-clerkship surveys were completed by 98 and 81 students, with response rates of 73.6% and 60.9%, respectively.

Results from the post-course survey (Table 2) indicated that 98% of 98 respondents strongly agreed or agreed that the variety of options (podcasts, videos, readings, etc.) offered in the course allowed for individualization of learning. Additionally, 93.8% of these respondents strongly agreed or agreed that the course was well organized, and 96% strongly agreed or agreed that the course load was appropriate for the allotted time. Nearly 96% of students strongly agreed or agreed that the virtual course improved their baseline understanding of fundamental OBGYN topics. When asked if the two virtual case discussions at the end of the course were beneficial in applying the knowledge they learned, 90% either strongly agreed or agreed that they were. Forty-one percent of students strongly agreed, and 45.9% agreed that the course enhanced their readiness to begin their core clerkship in OBGYN. When asked to compare the degree of learning achieved from the course to previous face-to-face courses or classes, 70.4% strongly agreed or agreed that this course reached a similar level of learning.

**Table 2:** Post-course survey results

Post-course survey	n = 133	%
The variety of teaching options offered in this course allowed for individualization of my learning (podcast, video, readings, etc.)		
Strongly agree	67	68.4
Agree	29	29.6
Neutral	2	2
Disagree	0	0
Strongly disagree	0	0
This course improved my baseline understanding of fundamental obstetrics and gynecology		
Strongly agree	54	55.1
Agree	40	40.8
Neutral	4	4.1
Disagree	0	0
Strongly disagree	0	0
I feel better prepared to start my core clerkship in obstetrics and gynecology after this course		
Strongly agree	41	41.8
Agree	45	45.9
Neutral	12	12.2
Disagree	0	0
Strongly disagree	0	0
The OB small group case discussion was beneficial in applying the knowledge I learned in the course		
Strongly agree	36	36.7
Agree	52	53.1
Neutral	4	4.1
Disagree	6	6.1
Strongly disagree	0	0
The GYN small group case discussion was beneficial in applying the knowledge I learned in the course		
Strongly agree	33	33.7
Agree	55	56.1
Neutral	4	4.1
Disagree	6	6.1
Strongly disagree	0	0
The degree of learning I achieved from this course was similar to previous face-to-face courses I've completed		
Strongly agree	25	25.5
Agree	44	44.9
Neutral	17	17.3
Disagree	8	8.2
Strongly disagree	4	4.1
I would complete this distance-learning course again if given the option between virtual or face-to-face		
Strongly agree	31	31.6
Agree	26	26.5
Neutral	17	17.3
Disagree	15	15.3
Strongly disagree	9	9.2
How likely are you to reference the information from the course during your clerkship?		
Very likely	48	49.0
Likely	39	39.8
Neutral	7	7.1
Unlikely	4	4.1
Very unlikely	0	0

Qualitative course assessment was based on free-text feedback regarding strengths, weaknesses, and suggestions to aid future course iterations. Example responses regarding course strengths included “I liked the freedom of the module to study as we saw fit.” “I liked the option of video vs. podcasts vs. chapters.” “I thoroughly enjoyed the flexibility of resources and the ability to choose those that are most beneficial to my style of learning.” and “I enjoyed seeing my peers and interacting with surgeons (my preceptors) from my future site. I also appreciated that we were given many options in regard to study materials and were able to approach each topic in the way that we learn best!”

Notable quotes from suggestions or weaknesses included “More group discussions.” “I believe increased difficulty in the cases would have been helpful to expand on the concepts and give the faculty something to truly ‘teach’ during those sessions.” and “More pointed objective questions. Questions for the Case groups were very broad and did not emphasize the salient takeaways.”

When assessing future projections in distance learning, 58.1% strongly agreed or agreed, whereas 9.2% strongly disagreed that they would complete this distance-learning course again if given the option between virtual and face-to-face learning. A cumulative 89% of students reported they were either very likely or likely to reference the information from the course during their clerkship. 

Results from the post-clerkship survey (Table 3) showed that 50.6% of 81 respondents strongly agreed or agreed that the OBGYN course helped prepare them for their OBGYN in-person clerkships. When answering the question, “The virtual course was helpful in preparing me for the OBGYN NBME exam,” 49.4% strongly agreed or agreed. 

**Table 3:** Post-clerkship results

Post-clerkship survey	N	%
The Ob/Gyn course was helpful in preparing me for my Ob/Gyn clerkship		
Strongly agree	8	9.9
Agree	33	40.7
Neutral	32	39.5
Disagree	7	8.6
Strongly disagree	1	1.2
The Ob/Gyn course was helpful in preparing me for the Ob/Gyn NBME exam		
Strongly agree	5	6.2
Agree	35	43.2
Neutral	20	24.7
Disagree	19	23.5
Strongly disagree	2	2.5
I referenced the resources provided in the course during my clinical rotation		
Very frequently	4	4.9
Frequently	13	16
Sometimes	26	32.1
Rarely	12	14.8
Never	26	32.1

When determining how useful the course materials were for students during their in-person clerkships, 20.9% of students reported they “very frequently” or “often” referenced the course resources. Finally, students were asked if they would recommend using the course in the future as preparation for in-person clerkships, and 41.9% of students either agreed or strongly agreed, while only 14.8% disagreed or strongly disagreed. 

Qualitative data collected from free-response questions in the post-clerkship survey identified what students found most and least helpful about the course. Some responses to “What did you find most helpful about the course after completing your OBGYN clerkship?” included the following: “Familiarization with the basics of OBGYN prior to starting the rotation,” “Providing an introduction and resource map to OB/GYN,” and “Allowed me to explore resources I would use during the actual clerkship to study.”

Students also referenced exposure to topics they had not previously covered; for example, “Reviewing fetal heart tracings. That’s not something we learned during pre-clerkship, so it made it easier to catch on when learning it again during our rotation,” and “Exposure to basic OBGYN material like normal labor, PPH, contraception, and STI screening. I feel like this wasn't covered much in pre-clerkship, so the CCIM material helped me to get a head start and look less dumb at the beginning of the rotation.”

Regarding what students felt was least helpful about the course in the post-clerkship survey results, many referenced the amount of time between course completion and their in-person clerkships. Some responses included “The time between the course material and the rotation made recall difficult. Also, the topics presented in the module, while very applicable within the context of the rotation, felt random during the actual course time.” and “The course was too long ago for it to have an impact on my clerkship.”

## Discussion

Our objective for this interim, virtual, self-paced, and guided clerkship preparation course was to fill the void while students were abruptly removed from their clinical rotations during the COVID-19 pandemic. While it is impossible to replace the gold standard of clinical learning through direct patient care, we endeavored to lend guidance and clinical direction so that students could return to their clinical experiences well-prepared. The responses from our surveys indicated that the course was beneficial, fostering individualized learning and promoting clerkship preparation. 

One major strength of this curriculum was the individualization of learning opportunities. We recognize that students vary in their learning preferences and that they largely dictate their own self-paced study plans while on clerkships while still maintaining clerkship content criteria [8]. By offering a spectrum of resources from previously vetted, high-quality textbooks, articles, podcasts, video clips, and practice questions, we not only supported our goal but created a succinct reference for students to use during their clerkships and while studying for the NBME exams. Another strength that students reported was the case discussions. While self-study allows students to interact with the material in an individualized fashion, it lacks a direct interface with experienced preceptors and the capability to apply their learned knowledge to a clinical case-based discussion. Students’ responses validated the benefit of this aspect. Finally, this course spanned four days, with only two scheduled events, again lending the opportunity for students to study at their own pace; this ability to create one’s schedule was especially critical during the COVID-19 pandemic as students were challenged with balancing their home lives and new schedules. Furthermore, although this course was created to complement the OBGYN clerkship, similar courses can be designed for other medical specialties using this structure.

We recognize the weaknesses of this course, which primarily relate to the inability to replace in-person clinical learning and the large timing gap between the completion of the course and the start of in-person clerkships. Additional limitations include the moderately small sample size of learners at a single institution, self-reported outcomes with potential recall bias, and the lack of controlling confounding variables, as the authors could not verify that the learners used the resources provided in the course exclusively without external independent content research. However, as a four-day virtual summary and preparedness course, we found that about half of the students felt the course prepared them for their in-person clerkship and helped them prepare for their NBME exam. Additionally, although most students thought they would reference the materials during their OBGYN clerkship, approximately 50% did. While most students found the course beneficial and felt well-prepared for their clerkships afterward, we need more data to validate whether this course did, in fact, meet this goal.

## Conclusions

Overall, a successful, short, interim, distance learning, virtual OBGYN clinical course was developed during a time of need. While the COVID-19 pandemic no longer restricts in-person clerkships, it catalyzed systemic change in medical education. This course has sustainable potential either as an introductory module for the OBGYN clerkship or to fill gaps for students who may miss clinical time due to illness or personal need. Additionally, this course structure can easily be adapted for other medical and surgical specialties.
